# Don’t Forget the Doctor: Gastroenterologists’ Preferences on the Development of mHealth Tools for Inflammatory Bowel Disease

**DOI:** 10.2196/mhealth.3987

**Published:** 2015-01-21

**Authors:** Trevor van Mierlo, Rachel Fournier, Richard Fedorak

**Affiliations:** ^1^Evolution Health Systems Inc.Toronto, ONCanada; ^2^Research Associate, Henley Business SchoolUniversity of ReadingOxfordshireUnited Kingdom; ^3^Faculty of Medicine and DentistryDepartment of MedicineUniversity of AlbertaEdmonton, ABCanada; ^4^The Centre of Excellence for Gastrointestinal Inflammation and Immunity ResearchEdmonton, ABCanada

**Keywords:** mHealth, adherence, concordance, compliance, shared decision making, therapeutic alliance, gastroenterology, IBD, ulcerative colitis

## Abstract

**Background:**

Inflammatory bowel disease (IBD) encompasses a number of disorders of the gastrointestinal tract. Treatment for IBD is lifelong and complex, and the majority of IBD patients seek information on the Internet. However, research has found existing digital resources to be of questionable quality and that patients find content lacking. Gastroenterologists are frontline sources of information for North American IBD patients, but their opinions and preferences for digital content, design, and utility have not been investigated. The purpose of this study is to systematically explore gastroenterologists’ perceptions of, and design preferences for, mHealth tools.

**Objective:**

Our goal was to critically assess these issues and elicit expert feedback by seeking consensus with Canadian gastroenterologists.

**Methods:**

Using a qualitative approach, a closed meeting with 7 gastroenterologists was audio recorded and field notes taken. To synthesize results, an anonymous questionnaire was collected at the end of the session. Participant-led discussion themes included methodological approaches to non-adherence, concordance, patient-centricity, and attributes of digital tools that would be actively supported and promoted.

**Results:**

Survey results indicated that 4 of the 7 gastroenterologists had experienced patients bringing digital resources to a visit, but 5 found digital patient resources to be inaccurate or irrelevant. All participants agreed that digital tools were of increasing importance and could be leveraged to aid in consultations and save time. When asked to assess digital attributes that they would be confident to refer patients to, all seven indicated that the inclusion of evidence-based facts were of greatest importance. Patient peer-support networks were deemed an asset but only if closely monitored by experts. When asked about interventions, nearly all (6/7) preferred tools that addressed a mix of compliance and concordance, and only one supported the development of tools that focused on compliance. Participants confirmed that they would actively refer patients and other physicians to digital resources. However, while a number of digital IBD tools exist, gastroenterologists would be reluctant to endorse them.

**Conclusions:**

Gastroenterologists appear eager to use digital resources that they believe benefit the physician-patient relationship, but despite the trend of patient-centric tools that focus on concordance (shared decision making and enlightened communication between patients and their health care providers), they would prefer digital tools that highlight compliance (patient following orders). This concordance gap highlights an issue of disparity in digital health: patients may not use tools that physicians promote, and physicians may not endorse tools that patients will use. Further research investigating the concordance gap, and tensions between physician preferences and patient needs, is required.

## Introduction

### Background

Inflammatory bowel disease (IBD) encompasses a number of disorders of the gastrointestinal tract, which are usually classified as Crohn’s disease (CD) or ulcerative colitis (UC). IBD is widespread, and it is estimated that as many as 2.2 million Europeans and 1.4 million Americans suffer from IBD [1]. With approximately 0.7% of the Canadian population diagnosed with the disease, Canada has one of the highest rates of IBD in the world [2,3].

Environmental, genetic, and intestinal microbial factors contribute to the chronic nature of the disease, which requires continuous medical treatment and frequent outpatient visits. However, effective treatment is available [4-6], and patients who do not take their medication have a five-fold risk of relapse than those who are adherent [7]. Despite the availability of treatment, there have been increases in hospitalizations for IBD, with significant economic costs [8,9], yet specific factors related to non-adherence in immunology are largely unknown [10].

As in other conditions, medication non-adherence in IBD is often noted as a cause of relapse and increased health care burden. For example, while adherence resulted in shorter hospital length of stay and lower inpatient costs among CD patients in one study [11], another discovered that when compared to adherent UC patients, those who were non-adherent incurred twice the inpatient costs and significantly higher health care costs [12].

As treatment for IBD is lifelong and variable, accurate patient education is critical. A recent survey found that the majority of IBD patients seek information on the Internet, but that information is of questionable quality [13]. In another US-based IBD clinic, it was found that over half of patients used the Internet to gather information, and Web-based resources ranked closely behind obtaining information from patients’ gastroenterologists [14]. A third study found that the quality of websites containing information on IBD varied widely, with most material being too difficult for patients to comprehend [15].

IBD experts generally agree that digital tools are a major resource for patients, but it is difficult for patients to determine which sites are accurate. While some resources may assist physicians, others may promote dangerous misunderstandings and misconceptions [16].

Successful digital tools require needs assessment of not only patients, but physicians who “prescribe” them. Achieving expert support and collaboration will be required to meet all stakeholder needs, and tool design needs to be strategic and based on theory. Design factors from the perspective of prescribing physicians have yet to be explored, and this paper is the first step in addressing that gap.

### Terminology and Definitions

Lack of medication adherence is a well-known, systemic issue in health care. However, despite decades of research into non-adherence, terminology describing the common phenomenon of patients not taking medication as directed remains inconsistent [17,18]. To complicate the matter, there is no consensual standard for what constitutes adherence or non-adherence, even within serious conditions [19]. For clarity, adherence definitions reported in recent immunology research are reproduced here.

### Medication Adherence

Patients are generally considered adherent if they take >80% of their prescribed dose regimen (prescribed time and dose) [7,17,20]. Studies estimate that in IBD, non-adherence rates vary from 40-60% [21-24]; however, some studies have shown non-adherence to be as high as 72% [20,25]. Following dose regimen is the responsibility of the patient, and the vast majority of IBD literature largely describes medication adherence as inadequate.

### Unintentional and Intentional Non-Adherence

Medication non-adherence is generally defined as “intentional” or “unintentional” [26-28]; however, “voluntary” or “involuntary” is also referred to in the literature. Intentional non-adherence occurs when patients purposely do not take their medication. Examples are patients taking a drug holiday, purposefully avoiding side effects, lack of perceived need or benefit, or avoidance of other factors. Unintentional non-adherence occurs when patients do not take their medication due to forgetfulness, poor comprehension, cost, inconvenience, or other factors attributed to busy lifestyles, work, or family commitments. Especially in IBD where treatment is complex, a patient’s relationship with medicine is often based on their individual beliefs and behaviors [29].

### Compliance

The terms “adherence” and “compliance” are often interchanged, however, they are very different constructs. Patients are compliant if they follow their doctor’s orders and act in accordance with dosing regimen [30]. Compliance is generally regarded as a negative term as it implies a paternalistic relationship, submission to authority, and a situation where the patient is a passive observer with no control [31].

### Concordance

“Concordance” is the newly accepted term replacing adherence and compliance. Concordance implies shared decision making and enlightened communication between patients and their health care providers, leading to an agreed treatment protocol [32].

### Patient-Centric Models of Care

A patient-centric model of care is a holistic approach that focuses on patients’ feelings about being ill, their ideas about what is wrong with them, the impact on their daily functioning, and expectations of treatment [33,34]. Shared decision making is an important part of the patient-centric model; however, medication concordance and prescribing occurs only after health professionals have a thorough understanding of environmental determinants surrounding the patient [35]. Calibration of medication can occur only in follow-up appointments where issues like dose regimen, side effects, and other issues can be empirically explored. Research indicates that digital applications can be designed to enable concordance among physicians, patients, and families to ensure that procedures and decisions follow individual patient need [36].

### IBD and Digital Treatment Programs

Compared to other chronic conditions, limited research has been conducted on IBD treatment through the Internet or mobile phone [37]. A reason for this may be attributed to the complexity and variability of the disease.

The majority of Web-based research has focused on irritable bowel syndrome (IBS). Several randomized controlled trials on IBS Web programs have shown effectiveness [38-40], however, IBS is a far less severe disorder that does not cause inflammation, ulcers, or other permanent damage to the bowel [41].

To date, two digital interventions for IBD have shown some promise. The first is an American intervention, which used a laptop and a device (Home Telemanagement Device). At 6-month follow-up, improvements in quality of life (QoL) and patient knowledge were found [42], but at 1-year follow-up the intervention proved to be ineffective [43].

The second, a European program (Constant Care) has shown promise for UC patients in Denmark and Ireland. At 1-year follow-up, there were noted improvements in QoL, patient knowledge, and decreased number of acute and routine visits [44]. However, the program is multifaceted and requires intensive participation by a number of stakeholders. Offering the program on a population level may require a reshaping of the health care system for IBD patients both legally and economically [45], and it is unknown if the program would work in a North American setting.

### Objectives

Gastroenterologists are the most common sources of information for IBD patients in North America [19]. The opinions and preferences of gastroenterologists on the utility of digital resources have not been examined, especially in a Canadian context.

To critically assess these issues and elicit feedback from experts, consensus was sought in a closed meeting among 7 Canadian gastroenterologists. The session included addressing past experiences with digital resources designed to improve medication adherence, attributes of digital tools that could benefit the physician-patient relationship, and the completion of a 12-item questionnaire (see Multimedia Appendix 1).

As the gastroenterologists in the study hold teaching positions, their insights and preferences are regularly disseminated to practitioners. Given the relatively small population of Canada, the gastroenterologists in this study have the potential to impact the gastroenterological community. As such, research questions were specifically formulated to investigate their specific views and needs:

RQ1. For the field of gastroenterology, what methodological approach to adherence should be used in the creation of digital devices?

RQ2. What attributes of digital tools will be supported by gastroenterologists?

RQ3. To create value in daily practice, how should digital tools be positioned to gastroenterologists, family physicians, and other professional stakeholders?

## Methods

In November 2013, 7 Canadian gastroenterologists participated in a 1-day Scientific Advisory Board meeting in Toronto, Ontario, which was sponsored by Ferring Pharmaceuticals Inc. (Canada). Discussion largely focused on medication non-adherence in Canadian IBD patients. Gastroenterologists were paid an honorarium for their participation, were made aware that the discussion was recorded and were advised that anonymized results may be used in an academic study.

The gastroenterology community in Canada is quite small; in 2007, it consisted of approximately 550 practicing gastroenterologists or internists [46]. As 5-8 participants are generally considered sufficient for an exploratory study with a homogeneous group [47-49], this convenience sample was a rare opportunity to collect insights from subject-matter experts in significant leadership positions.

Although gastroenterologists received a meeting agenda, the three research questions were not disclosed, as a primary concern was that their disclosure would shift discussions toward intervention design. The intention of the focus group was to allow gastroenterologists to freely explore, among each other, their perceptions of existing digital tools and how efficacious tools could be positioned. The discussion was also used as a means to position questionnaire content.

Facilitators strategically introduced links between non-adherence and digital tools several times during the meeting, and audio transcripts recorded discussions. At the onset of the meeting, gastroenterologists were advised that an anonymous follow-up questionnaire would be disseminated, and results would be collected, analyzed, and disseminated.

The questionnaire was based on existing peer-reviewed studies where survey instruments were designed to assess the impact of digital health information on the patient-physician relationship [50-52]. The 12-item questionnaire focused on medication non-adherence (three items plus one open-ended question) and perceived patient use of digital resources and physician need (seven items plus one open-ended question).

The Consolidated Criteria for Reporting Qualitative Research (COREQ) was used to describe the focus group process (Multimedia Appendix 2) [10]. Descriptive statistics were analyzed in SPSS version 19 for Mac.

## Results

### Themes

Themes emerging from gastroenterologists’ discussions centered on the pervasiveness of non-adherence, how shared decision making should be positioned, and the thematic nature of digital tools that can assist in communicating with patients (see Table 1).

### Methodological Approach to Addressing Non-Adherence With Patients

In the questionnaire, gastroenterologists were asked to rate the impact of non-adherence on a scale of 1-9, with 1 being not a factor and 9 being extremely important. Almost all (6/7) indicated that non-adherence was a barrier to treatment, with a median score of 6. On the questionnaire, one gastroenterologist noted that medication non-adherence was not a barrier in active disease, and another that non-adherence is higher in rectal therapies, especially enemas.

The 7 gastroenterologists were asked if voluntary or involuntary non-adherence was more challenging to address, or if both were weighed equally; 2 gastroenterologists indicated voluntary, 3 indicated involuntary, and 2 weighed both types of non-adherence as equally challenging to address.

When asked about patient interventions, almost all (6/7) would prefer digital tools that addressed a mix of compliance and concordance, and only one gastroenterologist supported the development of tools that focused on compliance. No gastroenterologists endorsed tools that only centered on concordance.

In the discussion, gastroenterologists expressed the difference between results in randomized controlled trials (RCT) and real-world settings, with compliance rates much higher in RCTs than in actual practice. Denial, or patients not accepting the diagnosis of IBD, was also identified as an issue.

Gastroenterologists noted that digital tools might be used only by patients who are already adherent, and those who are non-adherent may also be non-adherent with digital tool usage. Gastroenterologists were also forthright and generally agreed that patient focus groups could provide unique insights in digital tool criteria that gastroenterologists were not in a position to offer.

### Assessment of Existing Digital Tools

Over half of the gastroenterologists (4/7) reported that patients brought digital resources to a visit; 5 found them to be inaccurate and irrelevant, but 4 regularly refer their patients to specific resources.

Gastroenterologists generally agreed that what is missing is a respectable digital resource that is fact-based but not overly commercial. A gap identified is the apparent lack of peer-to-peer support tools that have been successfully utilized in other conditions, and that this type of interaction could be especially beneficial for younger people. However, this type of resource would need to be moderated by experts or other health care professionals with specific knowledge in the field.

**Table 1:** Gastroenterologist opinions on non-adherence, shared decision making, and digital assets.

Theme	Research question	Representative quotations
Non-adherence and shared decision making	RQ1	Patients don’t really care about full remission. They care about going from 20 to five bowel movements a day.
	RQ2, RQ3	That’s the problem. Everyone is compliant in the study [Randomized Controlled Trials]. You need real world data [which can be collected through digital tool usage data].
	RQ1	I think IBD takes a long time to get to grips with. If you have a heart attack, you can deal with it right away mentally. IBD in my practice takes months or years to accommodate and really understand. Young males are the worst. They take a decade to deal.
	RQ1	I think we should be concentrating equally on what the patient wants: a response as much as a remission rate. That’s going to give you a different set of numbers.
	RQ3	Gastroenterologist 1: Another factor with the younger patient is the rapport with the physician. That’s extremely important. How they connect. In other words, education for the physicians. Gastroenterologist 2: How do you achieve that in seven minutes?
	RQ2, RQ3	This is a huge thing [digital tools targeting non-adherence in IBD]. This is very, very ambitious. What’s to say that patients who have adherence problems aren’t going to have problems adhering to the [digital] program? It will always come back to the physician…you say you’re trying to offload the physician so there’s less work. But you’re talking about motivating the patient to become adherent, but that has to come via some sort of interaction. And usually, the best sort of interaction is in the physician’s office. If patients are going to be involved in this, then I think physicians have to be involved.
	RQ2, RQ3	I think also what you haven’t done as yet is that you need to have multiple patient focus groups to get their insight and to select people who would meet your non-adherent patient criteria. If you can ask them and get their feedback, they will provide data and insight that we [gastroenterologists] can’t offer…
Digital solutions for IBD		...tools are important. I’m more and more convinced that effective visual tools are the way to get them [patients] to do what you want, with me having to do less verbiage.
	RQ3, RQ1	It’s been done [Internet sites for IBD]. People have tried this…focusing on lifestyle modification. It looks good and when you think about it…but no one actually does [uses the program]. So I know from experience: I don’t actually use this great site!
	RQ3	We have all these disparate [Internet] tools, some bad—some great, that we don’t use. I don’t know how to bring those together in a better format.
	RQ3	I’m trying to teach them [patients] how to use it [enemas]. That’s why I’m using the YouTube video. If you take someone who’s 20, and say “here take this enema”, it spills on their sheets, it’s messy, it’s painful…A good cartoon, showing how to lay down, how to put a towel under yourself in case it leaks, and so on…that’s where practical things would be really valuable.
	RQ2, RQ3	I think you need to show that it’s a respectable site, and not commercial. There’s a plethora of info out there; you don’t want to just repeat it. We have sites already; you go on with a DIN number...and you navigate through that. Ideally, it would be best to go through a third party...You know you’re going to the right place.
	RQ1, RQ3	Gastroenterologist 1: What percentage of your patients spend time on the Internet? Gastroenterologist 2: 60% Gastroenterologist 3: I’d say 80%. They don’t want to have the disease. They don’t want IBD. The 20 year olds are wanting to ignore the disease. Gastroenterologist 4: They think you’re wrong. They don’t think they have it [IBD]. Gastroenterologist 1: It used to be a small number. Now it’s almost everybody.
		...if you start something like this [community-based digital tool], and you get everybody on board and excited, and the program peters out a year down the line, you have to be certain that you can keep the commitment.
		I think you’re on the right track [with digital tools targeting non-adherence]. The apps are so incredibly important these days...I equate it to this: if the patients have access to all that information, the challenge is how to keep that alive. How does that not fizzle out? A lot of sites have had a big fanfare, only to fizzle out.
		About peer-to-peer support, there’s always something lacking in young people and their ability to interact with peers with similar circumstances…that can work two ways, a crowd mentality can turn against you. Still, it’s something that’s never quite been there for IBD patients.
		I think we’ve [gastroenterologists] underutilized other health care professionals, like nurses, who could actually dialogue [on the Internet] with patients and could answer some of those ongoing questions and be that mother/father person on the site who is giving them that right information. We just don’t utilize that.

### Characteristics of Digital Tools: Content Type, Endorsement

All 7 gastroenterologists indicated that digital resources could benefit the physician-patient relationship, and 4 found that current digital resources did not increase their workload and patient misconceptions.

Gastroenterologists were asked which attributes of digital tools would increase their comfort level in regards to patient referral. All of them indicated that the inclusion of evidence-based facts was of extreme importance. None endorsed the inclusion of standardized text, and 4 wished to see patient-centric tools (see Table 2).

The 7 gastroenterologists were also asked to consider the importance of the source of the digital tool: 4 indicated that professional association or non-profit agency endorsement was important, and only 2 noted that publishing results from interventions was important. The one respondent who answered “other” indicated in the development of digital tools, endorsement by gastroenterologists was important.

A discussion ensued regarding patient Internet access. While one gastroenterologist felt that 60% of patients spent time on the Internet, another believed that the number was close to 80%, and a third remarked, “now it’s almost everybody”.

Gastroenterologists generally agreed that digital tools were important and could simultaneously help patients, aid in consultation, and save time. While a number of IBD digital resources exist, none has yet synthesized patient and expert need. Helpful resources may include practical information presented in visual format, such as videos explaining how to properly administer enemas. However, content would need to be continually refreshed and updated. See Table 3 for the summarized responses to our research questions.

**Table 2:** Gastroenterologist responses to survey question (N=7).

Question: If you were comfortable referring patients to a digital tool, which attributes would you support (select all that apply)?	Recommended, % (n)
Standardized test (eg, product monographs)	0 (0)
Patient-centric tools	57 (4)
Professional association or non-profit agency endorsement	57 (4)
Evidence-based facts	100 (7)
Published within the literature	29 (2)
Other	14 (1)

**Table 3:** Answers to research questions.

Research question	Answer
What methodological approach to adherence should be used in the creation of digital devices that will be used by gastroenterologists?	A mix of compliance and concordance, weighted toward compliance.
What attributes of digital tools will be supported by gastroenterologists?	Evidence-based facts and patient-centric tools. Endorsement by professional associations would be a benefit.
To create value in daily practice, how should digital tools be positioned to gastroenterologists, family physicians and other professional stakeholders?	Gastroenterologists will refer patients to tools that clearly explain IBD, how it effects patients differently, and the importance of medication maintenance.

## Discussion

### Principal Findings

Based on the qualitative approach and questionnaire results, specific content themes and design strategies emerged.

#### A Concordance Gap

Six gastroenterologists preferred tools that contained a mix of both compliance and concordance, and one supported interventions that focused on compliance. Given the general trend toward patient-centricity and shared decision making in medicine, it may be surprising to see that none of the gastroenterologists endorsed the creation of digital interventions that focus on concordance.

However, this focus on compliance may not be surprising when considering complexity of IBD, the variability in an individual’s course of disease, and the fact that IBD cannot be cured. In IBD, diversions from prescribed dose regimen will most likely result in disease flare. Given that IBD is a disabling condition, patients may confuse feeling better with remission, so consistency of physician recommended dose regimen is most likely key to maintaining healthy outcomes.

#### The Root of Non-Adherence

The impact of non-adherence and the need for compliance can be seen in other data. For example, the questionnaire asked gastroenterologists to rate how much of a barrier to treatment non-adherence was on a scale of 1-9 (with 1 being not a factor and 9 being extremely important), and the median score was 6. As one gastroenterologist notes, medication non-adherence is not a barrier in active disease. In the discussion, barriers such as denial and inability to administer medication (enemas, especially among young adults) was seen as a greater challenge.

From questionnaire data, voluntary and involuntary non-adherence are equally important and digital interventions need to focus on both. Further research may explore whether voluntary or involuntary non-adherence is prevalent in different demographics or specific stages of disease.

#### Shared Decision Making

All gastroenterologists supported digital tools that benefit the physician-patient relationship (the Therapeutic Alliance) through shared decision making. This may contradict their focus on compliance, which is a paternalistic approach. However, from discussions it becomes clear that a main cause of non-adherence is rooted in negative patient beliefs and issues such as denial and embarrassment. As such, traditional tools such as diaries and medication trackers, which have not proven successful for gastroenterologists or their patients, are not a priority.

#### Information Gap

The theme of non-adherence and patient beliefs is also reflected in gastroenterologists identifying the need for evidence-based tools that do not contain standardized text (eg, medical themed). Clearly, IBD is a personal disease and digital content should not be overly formal. However, in the questionnaire patient-centricity was only somewhat important (4/7). Such heterogeneous results can be difficult to interpret or draw broad conclusions. In discussions, gastroenterologists did note that patient focus groups could provide data and insights that gastroenterologists could not provide.

#### Current State of Digital Tools

As mentioned previously, current research indicates that the vast amount of IBD information on the Internet is questionable and too complex for the average patient [13-15]. This was confirmed in our study. Most digital information brought to the gastroenterologists by patients was seen as inaccurate (5/7). However, 4 gastroenterologists regularly refer their patients to specific resources and as mentioned previously, all believe that digital resources have the potential to improve the physician-patient relationship.

### Future Directions

Based on the insights from this Canadian study, current digital interventions in IBD are not meeting professional needs. Clearly, patients can benefit from learning the importance of adherence during all phases of disease, and the information must be presented in a clear, evidence-based format free from standard medical jargon.

Gastroenterologists appear to welcome the opportunity to refer patients to resources that promote dialogue that can facilitate shared decision making, provided that patients are provided with information that clearly outlines consequences related to medication non-adherence.

### Strengths and Limitations

The opportunity to engage with top Canadian gastroenterologists in a setting where facilitators encourage the free flow of ideas, personal experiences, and idea generation is rare. To our knowledge, this is the first study where a cohort of top Canadian gastroenterologists systematically explored their perceptions of, and needs for, digital tools.

The qualitative approach and the introduction of digital tools and adherence at specific intervals throughout the meeting formulated an environment where the research questions were indirectly addressed. The use of the brief 12-item questionnaire at the end of the session was also strategic as it was designed to seek individual gastroenterologist opinion after engagement with peers, and not a consensus. The questionnaire (Multimedia Appendix 1) is not IBD specific and can be used to explore the views of experts in other conditions.

All 7 gastroenterologists were Canadian academics and practitioners and represented several provinces and research centers. Results and outcomes are uniquely Canadian and may not be applicable to other geographic areas. For example, Canada’s Medicare system is provincially administered, and approximately 70% is publically funded while 30% comes from private sources [53]. Results from the American and Danish/Irish interventions described earlier may not be replicable in a Canadian setting, and digital interventions may need to be specifically developed for different health care settings and funding systems.

### Conclusions

According to Canadian gastroenterologists, they are eager to use digital resources that benefit the physician-patient relationship; however, current resources are largely inaccurate and unreliable.

Based on insights generated from the qualitative session and results from the questionnaire, gastroenterologists would prefer digital tools that focus on the importance of medication compliance and address both voluntary and involuntary non-adherence. Tools should be evidence-based, but patient-centric in that content is comprehensive and written in plain language (see Table 3).

Despite the trend of patient-centric tools that focus on concordance, gastroenterologists in this study would prefer digital tools that highlight compliance.

While this study gives insights into the needs and preferences of Canadian gastroenterologists, it does not address the needs and preferences of IBD patients. The results highlight the issue of disparity in digital health: patients may not use tools that physicians promote, and physicians may not endorse tools that patients need.

If digital tools are to be used, they need to be embraced by patients, their physicians, gastroenterologists, family members, and other health care stakeholders. Further research investigating concordance, and the digital gap between physician preferences and patient needs, is required.

## Multimedia Appendix 1

Gastroenterologist survey.

## Multimedia Appendix 2

Consolidated criteria for reporting qualitative studies (COREQ): 32-item checklist.

## Abbreviations

IBD: inflammatory bowel disease
QoL: quality of life
RCT: randomized controlled trial
