# Supplementary material for: Don’t Forget the Doctor: Gastroenterologists’ Preferences on the Development of mHealth Tools for Inflammatory Bowel Disease
Source: JMIR Mhealth Uhealth. 2015 Jan 21;3(1):e5. doi: 10.2196/mhealth.3987 (PMC4319145; doi:10.2196/mhealth.3987)
Supplement: Supplementary file 1 [file mhealth_v3i1e5_app1.pdf]

Multimedia Appendix 1: KOL Survey

Medication Non-adherence

1. On a scale of 1-10 how much of a barrier to treatment is non-adherence (1 being not a factor, 9 being extremely important)?

1 2 3 4 5 6 7 8 9

2. What is ***more*** challenging to address, **intentional/voluntary** (eg, drug holidays, avoiding side-effects) or **non-intentional/involuntary** (eg, forgetfulness, inconvenience) non-adherence?

[ ] intentional
[ ] non-intentional
[ ] both are equal

3. Adherence literature supports the development of interventions. Should the theme of interventions promote:

[ ] compliance (patients following specific treatment regimens)
[ ] concordance (Shared Decision Making)
[ ] a mix of both compliance and concordance

4. To identify factors of non-adherence in IBD we conducted a preliminary literature review using the Medical Subject Headings (MESH) terms: **Inflammatory Bowel Disease**, **adherence**, and **Pentasa**. Which additional keywords would you consider using?

____

____

____

Digital Resources (Internet, tablet and mobile phone)

5. Have you experienced patients bringing digital resources or information to a visit?

[ ] Frequently
[ ] Infrequently
[ ] Not at all
[ ] Not applicable

6. Have you ever experienced a patient brining inaccurate digital resources or information to a visit?

[ ] Yes
[ ] No
[ ] Not applicable

7. In your experience, are digital resources for patients generally accurate and relevant?

[ ] Frequently	[ ] Not at all
[ ] Infrequently	[ ] Not applicable

8. Are there specific digital resources that you refer patients to?

[ ] Yes (please list): ____
[ ] No
[ ] Not applicable

9. Is it possible for digital resources to benefit the physician-patient relationship?

[ ] Yes
[ ] No

10. The majority of the literature indicates that digital resources are burdensome for physicians (increasing workload and patient misconceptions). Do you find this to be true?

[ ] Yes
[ ] No
[ ] Not applicable

11. If you were comfortable referring patients to a digital tool, which attributes would you support? Please select all that apply.

[ ] standardized text (eg, product monographs)<br>[ ] patient-centric tools<br>[ ] professional association or non-profit agency endorsement	[ ] evidence-based facts<br>[ ] published within the literature<br>[ ] other: ______________________

12. Do you have any other comments regarding the questions above, adherence in IBD and/or digital adherence tools?

Sources used in the development of this anonymous questionnaire:

McMullan, M. (2006). Patients using the Internet to obtain health information: how this affects the patient-health professional relationship. *Patient Educ Couns, 63*, 24-28.

Murray, E., Lo, B., Pollack, L., Donelan, K., Catania, J., Lee, K., Turner, R. (2003). The impact of health information on the Internet on health care and the physician-patient relationship: national U.S. survey among 1.050 U.S. physicians. *J Med Internet Res, 5*(3), e17. doi: 10.2196/jmir.5.3.e17

Ivenson, S. A., Howard, K. B., & Penney, B. K. (2008). Impact of Internet use on health-related behaviors and the patient-physician relationship: a survey-based study and review. *J Am Osteopath Assoc, 108*(12), 699-671.

Ahmad F, Skinner HA, Stewart DE, Levinson W. Perspectives of Family Physicians on Computer-assisted Health-risk Assessments. J Med Internet Res 2010;12(2):e12.
