# Supplementary material for: Don’t Forget the Doctor: Gastroenterologists’ Preferences on the Development of mHealth Tools for Inflammatory Bowel Disease
Source: JMIR Mhealth Uhealth. 2015 Jan 21;3(1):e5. doi: 10.2196/mhealth.3987 (PMC4319145; doi:10.2196/mhealth.3987)
Supplement: Supplementary file 2 [file mhealth_v3i1e5_app2.pdf]

## Multimedia Appendix 2: Consolidated criteria for reporting qualitative studies (COREQ): 32-item checklist

| No Item | Guide questions / description |
|---|---|
| **Domain 1: Research Team and Reflexivity** | |
| **Personal Characteristics** | |
| 1. Interviewer/facilitator | The PI was TvM. Two employees from Ferring Pharmaceuticals (Canada) assisted in facilitating the focus group discussion. |
| 2. Credentials | The PI is a doctoral student with an MSc Community Health, an MBA, a Global Executive MBA, and a MSc Business and Management Research |
| 3. Occupation of PI | Chief Scientific Officer, Evolution Health Systems; Research Associate, Henley Business School, University of Reading |
| 4. Gender | Male |
| 5. Experiencing and training | The PI has been trained in both quantitative and qualitative research, has conducted past focus groups, has been a member of past focus groups, and has published extensively in the field of digital health. |
| **Relationship with Participants** | |
| 6. Relationship Established | The PI had no prior relationships with the study participants (KOLs: Key Opinion Leaders) prior to the study. |
| 7. Participant knowledge of the interviewer | Prior to the meeting participants were made aware of the PI's education and experience in digital health. |
| 8. Interviewer characteristics | The PI was introduced as an expert in digital health development, and not gastroenterology. KOLs were briefed on the purpose of the focus group, which was to examine their perceptions of existing digital tools for IBD patients, and their preferences for digital tools in a relaxed, conversational, and participatory environment. |
| **Domain 2: Study Design** | |
| **Theoretical Framework** | |
| 9. Methodological orientation and Theory | Focus group discussion followed by anonymous survey |
| 10. Sampling | Convenience sample |
| 11. Method of Approach | KOLs were recruited by Ferring Pharmaceuticals (Canada) through their extensive network into the Canadian Gastroenterology Community |
| 12. Sample Size | Seven Canadian KOLs were invited to participate in the study |
| 13. Non-participation | There were no drop-outs |
| **Setting** | |
| 14. Setting of data collection | The focus group was conducted in a conference room at a hotel in Toronto, Canada. The survey was distributed, completed, and collected the end of the meeting. |
| 15. Presence of non-participants | Apart from the PI and the two Ferring Pharmaceutical employees who assisted in the focus group, a professional transcriber attended the meeting. |

| | |
|---|---|
| 16. Description of the sample | KOLs held senior research and teaching positions at Canadian Universities, and their insights and preferences are regularly disseminated to practitioners. |
| 17. Interview guide | The PI and two Ferring employees pilot tested the focus group prior to the meeting. |
| 18. Repeat interviews | No repeat interviews were conducted. |
| 19. Audio / visual recording | The focus group was recorded and transcribed. |
| 20. Field notes | No |
| 21. Duration | The focus group was conducted on November 30, 2013, and focus group discussion was approximately 2 hours. |
| 22. Data saturation discussed? | No |
| 23. Transcripts returned | Transcripts were not returned to participants |
| **Domain 3: Analysis and Findings** | |
| **Data Analysis** | |
| 24. Number of data coders | Two data coders analyzed survey results (TvM, RF) |
| 25. Description of the coding tree | Focus group content was not coded |
| 26. Deviation of themes | Themes were derived from the data |
| 27. Software | SPSS version 19 for Mac was used to code survey results |
| 28. Participants checking | Participants did not provide feedback on findings |
| **Reporting** | |
| 29. Quotations presented | Quotations were presented to illustrate themes that emerged from the survey |
| 30. Data and findings consistent | There was consistency between the data presented and the themes |
| 31. Clarity of major themes | Major themes were presented in the findings |
| 32. Clarity of minor themes | Further research is required to uncover minor themes |
